# Unraveling Emerging Data on Lipoprotein(a)-Driven Cardiovascular Disease via Multiomics: A Review

**DOI:** 10.5334/gh.1540

**Published:** 2026-03-23

**Authors:** Szilard Voros, Michael R. Barnes, David Watson, Wess Boatwright, Anthony Lozama, Denise Yates, Jagat Narula, Santica Marcovina

**Affiliations:** 1 G3 Therapeutics, Midlothian, VA, USA; 2 King’s College London, London, UK; 3 Novartis Pharmaceutical Corporation, East Hanover, NJ, USA; 4 Biomedical Research (Novartis), Cambridge, MA, USA; 5 University of Texas Health Science Center at Houston, UTHealth Houston University of Texas, Houston, TX, USA; 6 Medpace Reference Laboratories, Cincinnati, OH, USA

**Keywords:** atherosclerosis, atherosclerotic plaque, coronary computed tomography angiography, systems biology

## Abstract

Evidence has shown that lipoprotein(a) (Lp[a]) is an independent, causal, genetic risk factor for cardiovascular disease (CVD) that promotes the progression of high-risk, vulnerable atherosclerotic plaque phenotypes. Systems biology integrates multiomics datasets to study linear and nonlinear relationships to enhance understanding of the molecular patterns of disease. One such example is the Genetic Loci and the Burden of Atherosclerotic Lesions (GLOBAL) study, which utilizes multiomics profiling to unravel the molecular signatures of Lp(a)-driven CVD. Using deep phenotyping of coronary atherosclerosis by coronary computed tomography angiography, whole-genome sequencing for genetic analysis, and evaluation of thousands of omics measurements and circulating biomarkers, it is possible to describe the atherogenic milieu associated with Lp(a)-driven CVD. By leveraging the multiomic evaluation of Lp(a)-driven coronary phenotypes, we can begin to translate these findings into real-world strategies for earlier recognition of distinct Lp(a)-driven CVD, which may contribute to improved risk mitigation strategies in clinical practice.

## Graphical Abstract

**Figure:**
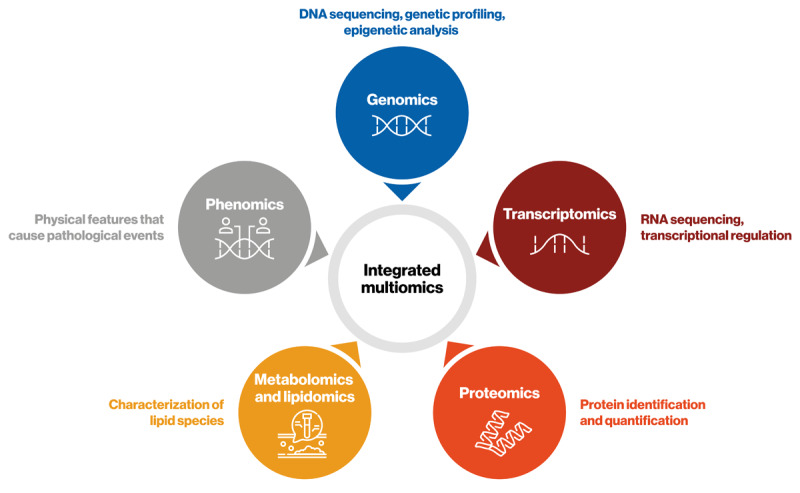


## Multiomics Analyses

Multiomics integrates analyses of numerous parameters, including DNA, RNA, and protein expression, to help unravel biological networks that contribute to the development and progression of disease. Analysis of multiomics biological big data can help to identify diagnostic and/or prognostic biomarkers of disease.

## Introduction

Elevated lipoprotein(a) (Lp[a]) levels, (>50 mg/dL or 105 nmol/L) (1,2), increase the risk of cardiovascular disease (CVD) and affect an estimated 1.5 billion people worldwide (3). Moreover, several studies have highlighted the role of Lp(a) in premature CVD and accelerated progression of CVD, as reviewed by Melita et al. (4). While the role of low-density lipoprotein-cholesterol (LDL-C) in atherosclerosis is well established, knowledge gaps remain on the wide-ranging roles of elevated Lp(a) in this setting (5). Importantly, Lp(a) is emerging as not just a biomarker but an important risk driver implicated in multiple pathophysiological processes that contribute to CVD (6). Indeed, several studies have reported that the CVD risk associated with elevated Lp(a) levels may exceed that of LDL-C on a per-particle basis (2,6,7). A meta-analysis by Willeit et al. showed that Lp(a) remained a predictor of cardiovascular risk even when LDL-C-attributable CVD risk was reduced with statin treatment (8), suggesting that elevated Lp(a) is an independent CVD risk driver regardless of LDL-C levels. In addition, evidence suggests that elevated Lp(a) increases the risk of major adverse cardiovascular events (MACE). A recent retrospective cohort study examined the risk of MACE in 16,419 individuals who underwent routine Lp(a) testing in the United States, and found that increasing Lp(a) concentration was independently associated with the incidence of MACE in individuals with and without atherosclerotic cardiovascular disease (ASCVD) at baseline (9). Furthermore, Wong et al. evaluated the impact of elevated Lp(a) in a pooled, multiethnic cohort of 27,756 individuals without ASCVD in the United States, reporting that compared with individuals with Lp(a) levels <50th percentile, those with Lp(a) ≥90th percentile had an adjusted hazard ratio of 1.46 (95% CI, 1.33–1.59) for ASCVD events, highlighting the importance of identifying patients with elevated Lp(a) in clinical practice (10).

Although the independent role of elevated Lp(a) in ASCVD risk has been robustly demonstrated (11), elevated Lp(a) has also been shown to act synergistically with inflammatory mediators to increase cardiovascular risk (12). Lp(a) exerts its proinflammatory effects via oxidized phospholipids (OxPLs), which activate endothelial cells and macrophages (13). Subsequent upregulation of adhesion molecules stimulates the recruitment of monocytes, fueling arterial wall inflammation (13). Inflammation itself is an established independent risk factor for ASCVD, and patients with elevated high-sensitivity C-reactive protein (hsCRP) and/or interleukin-6 levels have a higher risk of cardiovascular events compared with patients who have normal levels (14,15). Importantly, patients with both elevated Lp(a) and elevated inflammatory markers exhibit increased cardiovascular risk compared with patients presenting with either biomarker alone, emphasizing the synergistic relationship between Lp(a) and inflammation (14,15). As such, universal screening for Lp(a) and hsCRP among individuals at risk of ASCVD has been recommended as part of a holistic approach to CVD evaluation and risk assessment (16).

The magnitude of elevated Lp(a) as a CVD risk driver is recognized by multiple medical societies that recommend screening for Lp(a) in individuals with a personal and/or family history of elevated Lp(a) or premature CVD (2,17,18). In early 2024, in response to accumulating epidemiological data clarifying the relationship between elevated Lp(a) and CVD risk, the National Lipid Association (NLA) issued updated guidance stating that sufficient evidence exists to recommend universal Lp(a) testing for risk stratification at least once in a lifetime in adults (18). The NLA also recommends selective screening for Lp(a) in children <18 years of age if they have clinically suspected or genetically confirmed familial hypercholesterolemia, first-degree relatives with a history of premature ASCVD, history of ischemic stroke of unknown cause, or first-degree relatives with elevated Lp(a) levels (18). However, despite being an established, genetically determined, independent, causal risk driver for ASCVD, most patients with ASCVD in the United States are currently managed without knowledge of their Lp(a) levels (19). The Lp(a)HERITAGE study of patients with ASCVD reported significantly higher median Lp(a) levels in those <65 years of age compared with participants ≥65 years of age (20), which suggests that Lp(a) may be a driver of premature ASCVD. Women and younger patients tended to have higher levels of both LDL-C and Lp(a) in the Lp(a)HERITAGE study, reflecting the influence of both lipoproteins on premature ASCVD (20). Similarly, a recent meta-analysis of 100,540 patients concluded that elevated Lp(a) is associated with premature composite and individual ASCVD (21), and elevated Lp(a) was also shown to be independently associated with increased risk of acute coronary syndrome in people <45 years of age (22).

To better define the pathophysiology of Lp(a)-driven CVD, a greater understanding of the high-risk characteristics that arise in patients with elevated Lp(a) levels is needed. For instance, elevated Lp(a) is associated with the progression of low-attenuation coronary plaque phenotypes that increase the risk of myocardial infarction (MI) (23); however, further studies are needed to understand the potential causality between elevated Lp(a) and high-risk phenotypes such as heterogeneous atherosclerotic plaque, which is identified as partially calcified plaques (PCPs) (24), and high-risk features such as the napkin-ring sign (NRS; defined as low computed tomography [CT] attenuation in the plaque center surrounded by an area of higher attenuation), which is identified by contrast-enhanced coronary CT angiography (25). Multiomics studies integrate analyses of numerous parameters, including DNA variants, gene expression (RNA), protein expression, and lipidomics, which provide insights into the biological networks that contribute to the progression of disease and associated clinical phenotype (26). The Genetic Loci and the Burden of Atherosclerotic Lesions (GLOBAL) study (NCT01738828) generated deep phenotyping of coronary atherosclerosis by coronary computed tomography angiography (CCTA) coupled with extensive and comprehensive multiomic profiling along the central dogma of biology, which describes the DNA > RNA > proteins > phenotypes > clinical outcomes spectrum. Deep multiomic profiling included whole genome sequencing, whole genome methylation analysis, whole transcriptome sequencing, proteomics, metabolomics, lipidomics, and the evaluation of a comprehensive suite of conventional biomarkers. This dataset is being used to unravel the complexities of CVD, including the unique condition of Lp(a)-driven CVD (Graphical Abstract) (25).

Herein, key Lp(a) research questions are described, with a focus on gaining a granular understanding of the phenotypic features of Lp(a)-driven CVD to support more efficient identification of high-risk patients and potentially those at risk of premature and/or recurrent cardiovascular events in clinical practice.

## Coronary Atherosclerosis

### Initiation, progression, and clinical spectrum of coronary atherosclerosis

**Figure 1:**
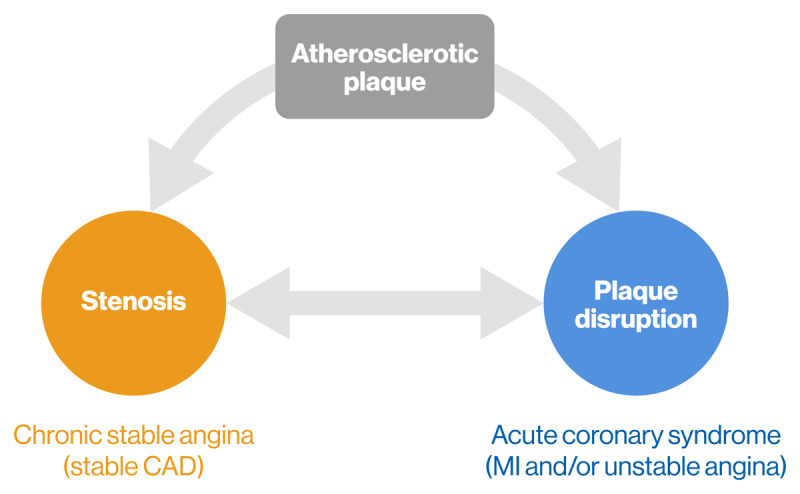
**Atherosclerotic plaque progression**. Atherosclerotic plaques grow and develop before taking one of two clinical pathways. Abbreviations: CAD, coronary artery disease; MI, myocardial infarction.

Lipids and lipoproteins are central components of atherosclerosis, and all apolipoprotein B100 (apoB)-containing lipoprotein particles are atherogenic (27). As coronary atherosclerosis progresses, plaques grow, mature, and eventually take one of two clinical pathways: (1) progressive luminal stenosis, resulting in hemodynamic compromise, decreased fractional flow reserve, ischemia, and finally, chest pain in the form of chronic stable angina; (2) plaque disruption, either by rupture or erosion, which leads to thrombosis and acute coronary syndrome, characterized by the occurrence of MI and/or unstable angina. Importantly, some interplay between these pathways may be observed when a ruptured plaque progresses, resulting in stenosis and ischemia (Figure 1).

#### Lp(a) in the initiation of atherosclerosis

Lp(a) is composed of a single apoB lipoprotein covalently bound via a disulfide bond to apolipoprotein(a) (apo[a]) (28). Apo(a) is encoded by the *LPA* gene, which contains 10 kringle 4 (KIV) subtypes (KIV_1_–KIV_10_) derived from plasminogen (29). Each apo(a) allele contains 2 to >40 copies of KIV_2_, resulting in over 40 different apo(a) isoform sizes across the global population (30). Apo(a) isoforms can be categorized as small (low-molecular weight [LMW]) or large (high-molecular weight [HMW]), defined herein as <24 or ≥24 KIV_2_ repeats, respectively (31), considered to be distinct entities. Indeed, data suggest that apo(a) isoform size is largely inversely correlated with Lp(a) concentration, whereby LMW apo(a) isoforms are associated with higher levels of circulating Lp(a) compared with HMW apo(a) isoforms (32). Moreover, a long-term observational study of 116 patients with early-onset coronary heart disease (CHD) reported that the LMW apo(a) phenotype was the most important risk factor for the development of MI (33).

Lp(a) exerts notable proatherogenic, prothrombotic/antifibrinolytic, and proinflammatory influences that contribute to the development and progression of atherosclerosis (13). In addition, studies have identified Lp(a) as the primary transporter of OxPLs in human plasma, which are known to orchestrate a myriad of proinflammatory effects (13). Lp(a)-induced inflammation leads to endothelial dysfunction, which results in barrier disruption and passage of Lp(a) through the protective endothelial layer of the arterial wall (13). Lp(a) also carries cholesterol, which may directly contribute to the initiation of atherosclerosis (34).

#### Lp(a) in the progression of atherosclerosis and the manifestation of cardiovascular events

Lp(a) drives CVD risk by promoting the progression of vulnerable plaque phenotypes with a high propensity for rupture (35). Interestingly, van Dijk et al. utilized immunohistochemical staining to visualize apo(a) in early and late atherosclerotic lesions, with particular abundance observed in the necrotic core of ruptured plaques (36). This observation is clinically relevant, given that ruptured plaques are associated with atherothrombotic events, including MI (37). Accordingly, Yu et al. identified an association with future MI risk in patients with elevated Lp(a) (≥50 mg/dL) and low-attenuation coronary plaques measured via CCTA; however, the causality of Lp(a) in MI was not demonstrated in this study (23).

As a noninvasive imaging technique, CCTA can be used to measure overall plaque burden in Lp(a)-driven CVD and phenotypic features of individual plaques and their progression over time (35). Importantly, CCTA may be superior to invasive coronary angiography for identifying phenotypic features of plaques (38) and thus has the potential to be an important strategy for the identification of high-risk patients or those at risk for premature events who may benefit from pharmacological Lp(a)-lowering therapy in the future (39).

**Figure 2:**
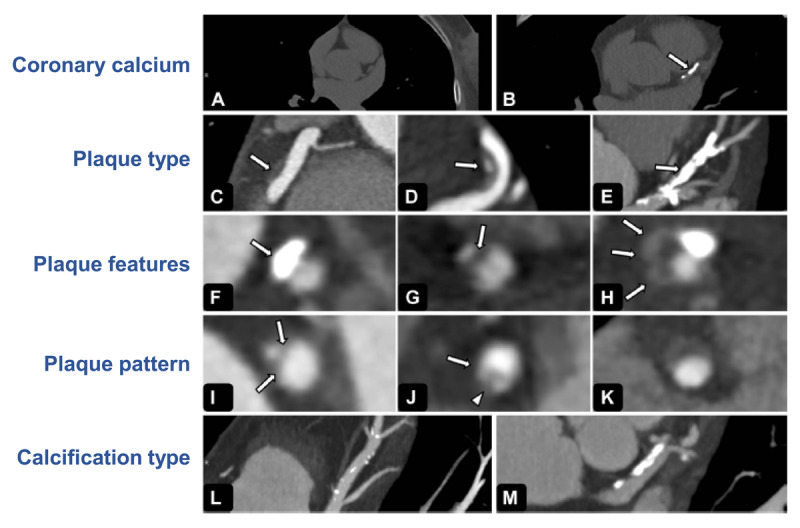
**Plaque analysis by CCTA**. To assess the **(A)** absence or **(B)** presence of coronary artery calcium. Plaque type is classified as noncalcified **(C)**, partially calcified **(D)**, or calcified plaque **(E)**. Plaque features are classified as positive remodeling (panel **(F)** shows external remodeling of calcified plaque), **(G)** low-attenuation plaque, and **(H)** positive remodeling. Plaque pattern refers to noncalcified regions of plaques and is classified as **(I)** homogeneous, **(J)** heterogeneous, or **(K)** napkin-ring sign noncalcified plaque with enhancing ring surrounding a low-attenuation core. Calcification type is classified as **(L)** spotty (calcified lesions <3 mm) or **(M)** large (calcified lesions >3 mm). Abbreviation: CCTA, coronary computed tomography angiography. Reprinted from Voros S, Maurovich-Horvat P, Marvasty IB, Bansal AT, Barnes MR, Vazquez G, et al. *J Cardiovasc Comput Tomogr*. 2014;8(6):442–451. https://doi.org/10.1016/j.jcct.2014.08.006 and reused in accordance with the Creative Commons Attribution (CC BY-NC-ND 4.0 DEED) license.

Figure 2 depicts atherosclerotic plaque phenotype analysis by CCTA, published by Voros et al. (25). This technique demonstrates the utility of identifying multiple plaque phenotypes and monitoring features that could indicate a high risk of progression and/or rupture. A 2022 study that utilized CCTA to analyze plaques in 191 patients with advanced stable coronary artery disease (CAD) reported that patients with elevated Lp(a) levels (≥70 mg/dL) experienced accelerated progression of low-attenuation coronary plaque volume at 12 months versus baseline compared with patients with lower Lp(a) levels (35). In addition, a cohort study of 500 patients with acute coronary syndrome showed that those with Lp(a) levels >30 mg/dL had a higher prevalence of lipidic plaque at the site of stenosis (67% vs 27%; *P* = 0.02) and higher prevalence of thin-cap fibroatheroma (38% vs 10%; *P* = 0.04) compared with patients with Lp(a) levels <30 mg/dL (40). Furthermore, a recent study by Nurmohamed et al. found that in 267 patients with suspected CAD who underwent CCTA at baseline and after 10 years, those with Lp(a) levels ≥125 nmol/L had a higher percent atheroma volume compared with patients with Lp(a) levels <125 nmol/L (6.9% vs 3.0%, respectively; *P* = 0.01). Increasing Lp(a) levels were also associated with increased presence of low-density noncalcified plaque (NCP), and, since calcification is thought to play a role in plaque stabilization (24), taken together, these data suggest that elevated Lp(a) may drive the progression of high-risk plaques over time (41).

### Elucidating a Holistic Understanding of Lp(a)-Associated Pathogenicity

#### Animal models

To date, a comprehensive understanding of Lp(a)-associated pathogenicity has been hampered by the absence of robust animal models to study the mechanistic factors underlying the role of Lp(a) in CVD (13). Lp(a) levels are generally <20 mg/dL in established human apo(a) transgenic mouse and rabbit models of atherosclerosis, which is below the level considered to increase cardiovascular risk in humans; furthermore, transgenic animal models can only express one isoform of apo(a), which does not reflect the wide range of isoform sizes that exist in humans (42). Nonetheless, some progress has been made; Assini et al. recently reported a sex-specific exacerbation of atherosclerosis in transgenic mice expressing pathogenic levels of fully human Lp(a) (43). The authors found that elevated Lp(a) led to a more severe atherosclerotic disease phenotype in female versus male mice (43), which may provide us with clues as to how CVD manifests in humans, more specifically in women versus men.

#### Omics approaches

Historically, researchers have used tools such as Mendelian randomization and meta-analyses to provide evidence for the association between Lp(a) and CVD (2). Building on these data, multiomics studies integrate analyses of numerous parameters, including DNA, RNA, and protein expression, to help unravel biological networks that contribute to the development and progression of disease (Graphical Abstract) (26). While each omics dataset offers a glimpse into disease pathogenesis, highlighting associations rather than direct causality, integrating complex omics data allows us to amass a deeper understanding of the factors driving disease pathology, which may provide a significant step toward the validation of existing data that have identified the causal role of Lp(a) in CVD (26,44,45).

To date, several studies have utilized multiomics approaches to further our understanding of CVD at the molecular level (46). A study by Chen et al. looked at the association between genetic variants and modified protein levels, reporting the identification of multiple protein quantitative trait loci and providing insights into integrated networks of genetic variants, associated proteins, and resulting CVD traits (47). Development of cardiovascular-focused repositories of omics data has been somewhat slower than in other fields, such as cancer. However, both general and specialist resources now exist, as reviewed by Vakili et al. (48). The American Heart Association’s Precision Medicine Platform (https://pmp.heart.org) facilitates researcher entry to controlled-access cardiovascular datasets for cloud-based analyses, while HeartBioPortal (49) (www.heartbioportal.com) is a publicly available web application that integrates existing CVD-related omics datasets to provide intuitive visualization and analysis for both expert and nonspecialist users.

#### The GLOBAL study

**Figure 3:**
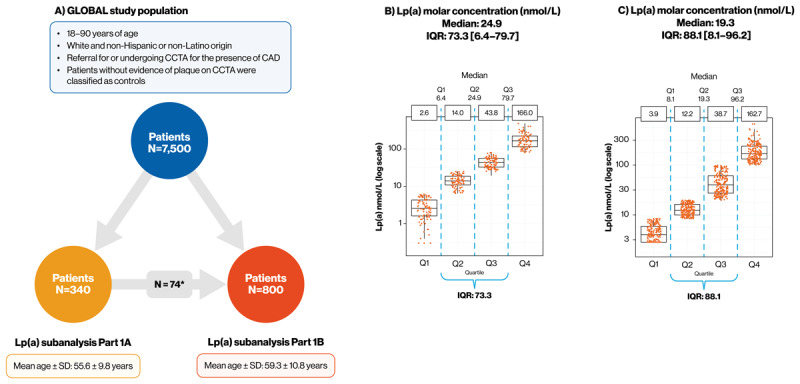
**The GLOBAL study. (A)** Patient population in the GLOBAL study and the Lp(a) subanalysis of the GLOBAL study; **(B)** Lp(a) distribution in Part 1A; **(C)** Lp(a) distribution in Part 1B. *Part 1B included 74 patients who were enrolled in Part 1A. Abbreviations: CAD, coronary artery disease; CCTA, coronary computed tomography angiography; GLOBAL, Genetic Loci and the Burden of Atherosclerotic Lesions; IQR, interquartile range; Lp(a), lipoprotein a; Q, quartile.

The GLOBAL study (NCT01738828) is an analysis of multiomic data from 7,500 patients referred for cardiac CT (Figure 3A), collected between 2012 and 2014. The study comprised three parts, each with independent discovery and validation cohorts (six analysis cohorts overall), with each patient allocated to one of the cohorts a priori (25). The discovery approach in the GLOBAL study was built on three key pillars: (1) deep phenotypic assessment by CCTA to quantify different aspects of the coronary vasculature and plaque morphology; (2) multiomics profiling of patient blood samples to understand the contribution of DNA, RNA, proteins, lipids, and other small molecules to CVD; (3) integration of CCTA phenotyping and multiomics data using bioinformatics to delineate the biological networks underlying CVD (25). Biological samples from enrolled patients underwent multiomics analyses as well as evaluation of a suite of conventional clinical biomarkers related to ASCVD and associated risk factors. In addition, CCTA was performed on all individuals and analyzed to establish the prevalent atherosclerotic plaque phenotypes in the subset of patients with elevated Lp(a) (25).

The Lp(a) subanalysis of the GLOBAL study, consisting of two parts, seeks to characterize the coronary plaque features associated with Lp(a)-driven CAD and to explore whether Lp(a)-driven CVD represents a distinct, high-risk cardiovascular phenotype (Figure 3A) (50). Part 1A enrolled 340 patients (median Lp[a]: 24.9 nmol/L; Figure 3B) and assessed associations between Lp(a) measurements and qualitatively assessed coronary atherosclerosis, luminal stenosis, and plaque type. The dependence of associations between Lp(a) measurements and plaque type on apoB was also assessed. In addition, the study evaluated the causality of Lp(a) on plaque type and aimed to characterize the molecular pathways mediating the atherogenicity of Lp(a) and LDL-C particles. Part 1B enrolled 800 patients (median Lp[a]: 19.3 nmol/L; Figure 3C) and aimed to determine whether Lp(a) levels are associated with coronary artery calcium score, quantitatively assessed plaque burden and composition, luminal stenosis, and the presence or absence of myocardial infarct and MI, including the extent and size of myocardial infarct.

### Key Research Questions and Preliminary Evidence to Date

A dedicated subanalysis of the ongoing GLOBAL study aims to answer multiple questions to define criteria by which high-risk patients with elevated Lp(a) can be identified in real-world practice. These patients may benefit from cardiovascular risk mitigation strategies, including Lp(a)-lowering therapies if they become available, which may help reduce the incidence of cardiovascular events and improve patient outcomes.

#### 1. Does Lp(a)-driven CVD represent a different, potentially higher-risk phenotype versus non–Lp(a)-driven CVD?

To understand whether Lp(a)-driven CVD represents a potentially higher-risk phenotype compared with non–Lp(a)-driven CVD, it is necessary to evaluate multiple factors such as plaque type, plaque features, and plaque pattern, as well as the degree of luminal stenosis, overall plaque burden, and lesion-level hemodynamic compromise. In particular, research is needed to fully elucidate the association between Lp(a) and coronary plaque phenotypes, including PCP, NCP, and calcified arterial plaque (CAP). Evidence suggests that calcification plays a role in plaque stabilization, which leads to reduced inflammation and may decrease the risk of cardiovascular events; therefore, the prevalence of PCP compared with CAP may indicate poor prognosis in patients with CVD (24). Importantly, PCP was shown to be associated with an increased risk of MACE compared with CAP or NCP in patients with CAD (51).

#### 2. Is the development of Lp(a)-driven coronary atherosclerosis different from non–Lp(a)-driven coronary atherosclerosis?

The initial event that stimulates coronary atherosclerotic plaque development is the retention of apoB-containing lipid and lipoprotein particles in the artery wall (52). With the progression of atherosclerosis, retention of lipids and lipoproteins is followed by the infiltration of inflammatory and smooth muscle cells into the plaque, which results in calcium deposition (53) and can be identified by the appearance of PCP on cardiac CT. This is when plaques may be more vulnerable to rupture than NCP or CAP (24). With healing and involution of the plaque, continued calcification is observed, and more stable CAPs are established (24). Accordingly, based on cardiac CT imaging, the traditional progression of coronary atherosclerotic plaque driven by LDL-C particles follows the sequence of NCP, to PCP, and finally, CAP. Whether Lp(a)-driven coronary atherosclerosis follows the same path or potentially a different path is currently being assessed.

#### 3. Are there patients with elevated Lp(a) who have high cardiovascular risk and may particularly benefit from Lp(a)-lowering therapies in the future?

**Figure 4:**
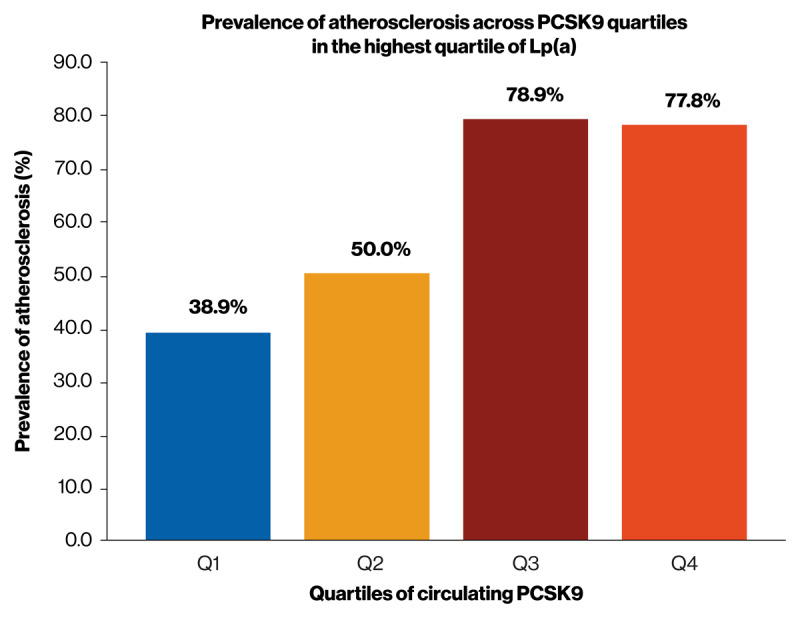
**Published data from the GLOBAL study**. Published data from Part 1A of the Lp(a) subanalysis of the GLOBAL study showed that increasing levels of circulating PCSK9 were associated with higher levels of circulating Lp(a), and this was associated with a high prevalence of atherosclerosis. For patients within the highest Lp(a) quartile, the prevalence of coronary atherosclerosis across PCSK9 quartiles 1–4 was 38.9%, 50.0%, 78.9%, and 77.8%, respectively. Abbreviations: GLOBAL, Genetic Loci and the Burden of Atherosclerotic Lesions; Lp(a), lipoprotein a; PCSK9, proprotein convertase subtilisin/kexin type 9. Reprinted from Brown BO, Watson DS, Boatwright W, Dayspring T, Barnes B, and Voros S. *J Clin Lipidol*. 2024;18(4):e577–e578 DOI: https://doi.org/10.1016/j.jacl.2024.04.117 (55), with permission from Elsevier.

To identify patients with elevated Lp(a) at the highest risk of cardiovascular events, we must further our understanding of predictive clinical features and/or blood-based measurements that may signify increased risk. Published data from Part 1A of the Lp(a) subanalysis of the GLOBAL study showed that increasing levels of circulating proprotein convertase subtilisin/kexin type 9 (PCSK9) were associated with higher levels of circulating Lp(a), which in turn was associated with high prevalence of atherosclerosis. When patients were grouped into Lp(a) and PCSK9 quartiles, the prevalence of coronary atherosclerosis increased sequentially across PCSK9 quartiles for patients with the highest Lp(a) levels (Figure 4) (31). In addition, the prevalence of both PCP and NRS, which is associated with advanced atherosclerotic lesions (54), was highest in patients within the highest quartile of Lp(a) and the highest quartile of serum PCSK9, potentially representing a high-risk group of patients with elevated Lp(a) in clinical practice (31). In addition, the association between PCSK9 and increasing Lp(a) levels was apparent for LMW apo(a) isoforms but not for HMW apo(a) isoforms (31). A retrospective analysis of 116 patients with premature CHD reported similar findings showing that the presence of the LMW apo(a) isoform resulted in a higher level of circulating PCSK9Lp(a) complexes compared with HMW apo(a) isoforms (33). Overall, the results of this study reiterate the importance of increased Lp(a) levels and, more specifically, the LMW apo(a) phenotype in patients with premature CHD. Building on these data, the Lp(a) subanalysis of the GLOBAL study aims to uncover likely causes of premature CAD and consequences to provide a robust rationale to identify those most at increased risk of premature events resulting from Lp(a)-driven CVD and to enhance screening.

#### 4. Are patients with elevated Lp(a) and with low, or at-goal, apoB and/or LDL-C levels at increased cardiovascular risk?

Published studies have identified Lp(a) as an independent CVD risk driver in patients with low LDL-C levels (56). The Lp(a) subanalysis of the GLOBAL study aims to establish the precise impact of elevated Lp(a) in CVD when other lipids and/or lipoproteins are at their optimal levels. For instance, do patients with elevated Lp(a) and low, or at-goal, apoB levels remain at increased cardiovascular risk, and is there a high prevalence of atherosclerosis in patients with elevated Lp(a) who are in the lower quartiles of apoB/LDL-C levels? Answers to these questions, which are under active investigation in the Lp(a) subanalysis of the GLOBAL study, will help to clarify the role of Lp(a) as an independent driver of CVD risk.

#### 5. Which features and measurements of Lp(a) are most informative about different aspects of cardiovascular risk?

Lp(a) particles comprise multiple elements, including apo(a), apoB, cholesterol, and OxPLs (57). Still, the features and measurements of Lp(a) that are most informative about different aspects of CVD remain to be elucidated. The Lp(a) subanalysis of the GLOBAL study aims to analyze a range of measurements related to Lp(a) to provide insight into which features are most closely associated with the phenotypic manifestations of Lp(a)-driven CVD.

#### 6. Does apo(a) isoform size make a difference to the atherogenicity of Lp(a) particles?

The Lp(a) subanalysis of the GLOBAL study will evaluate the impact of apo(a) isoform size on cardiovascular risk. Although data suggest that small apo(a) isoforms are associated with higher circulating levels of Lp(a), increased risk of ASCVD, and increased risk of premature cardiovascular events (32,33,58,59), the relative contribution and potential causality of small versus large apo(a) isoforms to different aspects of coronary atherosclerosis, for example, plaque type and plaque burden, requires further investigation. Nonetheless, routine measurement of Lp(a) levels can help to further our understanding of Lp(a) as a driver of cardiovascular risk.

## Conclusions

Evidence demonstrates that Lp(a) contributes to both the initiation and progression of atherosclerosis and that elevated Lp(a) levels are associated with high-risk plaque phenotypes. Going forward, a granular understanding of Lp(a)-driven CVD will be achieved by identifying the high-risk features that are most strongly associated with poor clinical outcomes. By utilizing omics technologies and advanced data analytics, deep molecular profiling and real-world phenotypes can be connected to provide greater insight into the features of high-risk disease. In addition, management strategies may be tailored to the range of patients with Lp(a)-driven CVD in real-world practice, including those with high-risk plaque phenotypes and those at risk of premature or recurrent cardiovascular events. The dedicated subanalysis of the Lp(a) cohort in the GLOBAL study dataset is ongoing and aims to provide a comprehensive insight into Lp(a)-associated pathogenicity to enhance clinical recognition of this at-risk population. The insights gained from the subanalysis of the GLOBAL study and other multiomics studies, including deep phenotyping by CCTA, may contribute to redefining risk in patients not considered typically high risk and identify patients who may derive particular benefit from Lp(a)-targeted therapies should they become available in the future.
